# COVID-19 and Influenza Co-Infection: Report of Three Cases

**DOI:** 10.7759/cureus.9852

**Published:** 2020-08-18

**Authors:** Balraj Singh, Parminder Kaur, Ro-Jay Reid, Fayez Shamoon, Mahesh Bikkina

**Affiliations:** 1 Hematology/Oncology, Saint Joseph's University Medical Center, Paterson, USA; 2 Cardiology, Saint Joseph's University Medical Center, Paterson, USA; 3 Internal Medicine, Saint Joseph's University Medical Center, Paterson, USA; 4 Cardiology, Saint Joseph's University Medical Center, Patterson, USA; 5 Cardiology, Saint Joseph's University Medical Center, Paterson, USA

**Keywords:** coronavirus, covid-19, influenza, severe acute respiratory syndrome coronavirus 2, sars-cov-2, co-infection, flu, influenza virus type a, influenza virus type b, influenza-like illness

## Abstract

Influenza and coronavirus disease 2019 (COVID-19) are both contagious respiratory illnesses, but they are caused by different viruses. COVID-19 pandemic is caused by a novel virus - severe acute respiratory syndrome coronavirus 2 (SARS-CoV-2). Influenza is an infectious respiratory disease, caused by influenza A and influenza B viruses. We describe the three cases of influenza and COVID-19 co-infection.

## Introduction

Coronavirus disease 2019 (COVID-19) originated in the Huanan South China Seafood Market in Wuhan. It has posed a global health threat. COVID-19 can present with a spectrum of clinical manifestations including fever, myalgia, cough, dyspnea, and less frequently headache, diarrhea, nausea, and vomiting. Although respiratory symptoms predominate, multiple organ dysfunction may also occur with COVID-19. Coagulopathy has been found as a prominent feature of COVID-19 and severe coagulation dysfunction may be associated with poor prognosis [1-3]. Neurological and cardiovascular complications are also common in COVID-19 patients. No effective treatment has yet been established.

## Case presentation

**Figure 1:**
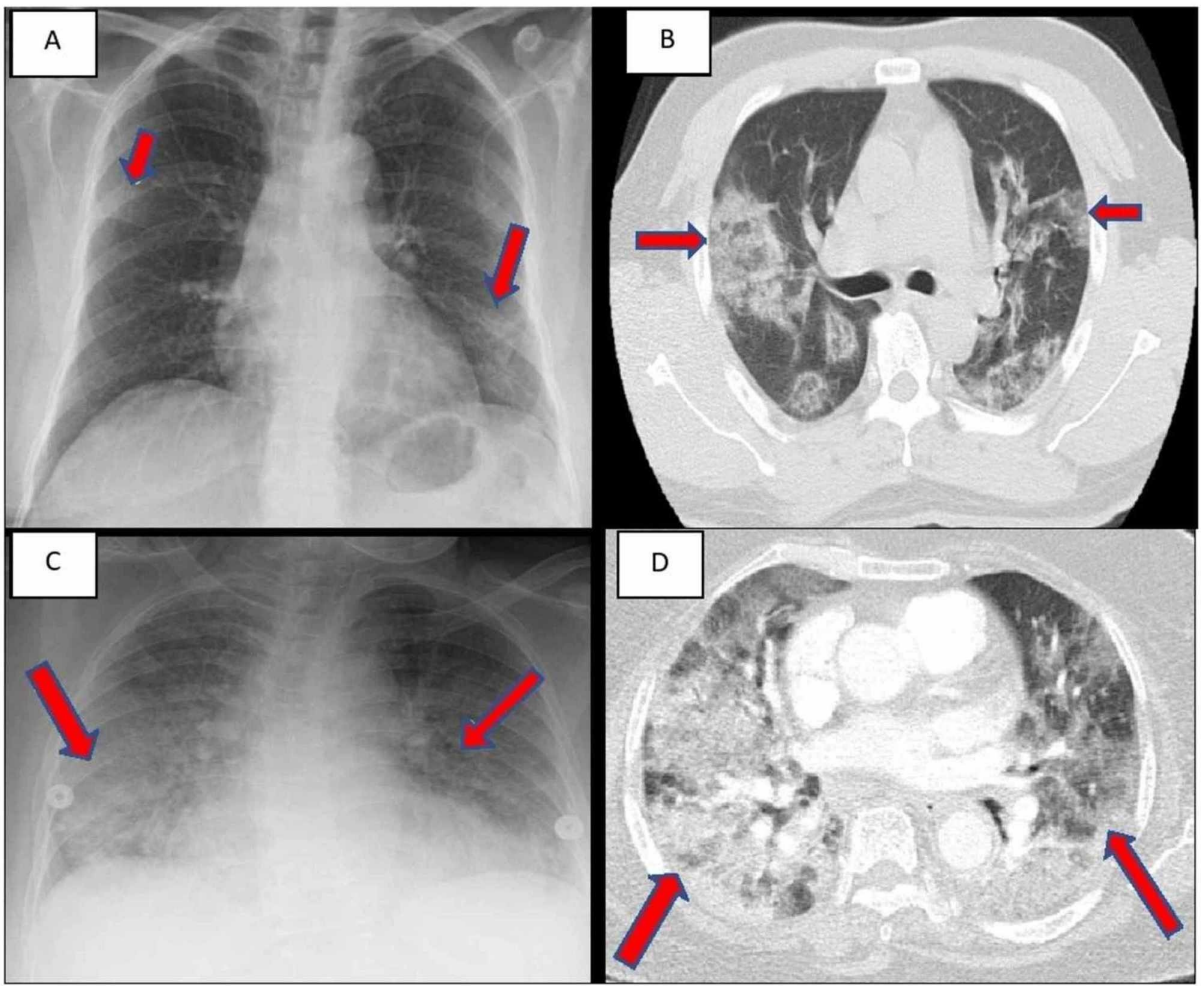
(A) CXR of case 1 showing bilateral patchy infiltrates. (B) CT chest of case 1 showing diffuse scattered areas of ground-glass and mixed attenuating opacities. (C) CXR of case 2 showing multi-lobar infiltrates. (D) CT chest of case 2 showing diffuse bilateral ground-glass infiltrates. CXR: Chest X-ray

The mean age was 59.6 years (range 47-71 years) and 2/3 were female. Two were Hispanic and one was Pilipino. Comorbidities included hypertension and diabetes mellitus. COVID-19 was diagnosed by nasopharyngeal swab reverse-transcription polymerase chain reaction (RT-PCR) and influenza by rapid antigen assay. Two patients had influenza type B and one had influenza type A. The presenting sign and symptoms were cough, fever, shortness of breath and myalgia. Chest X-ray (CXR) and computed tomography (CT) of the case 1 and case 2 are shown in Figure 1A-1D.

**Figure 2:**
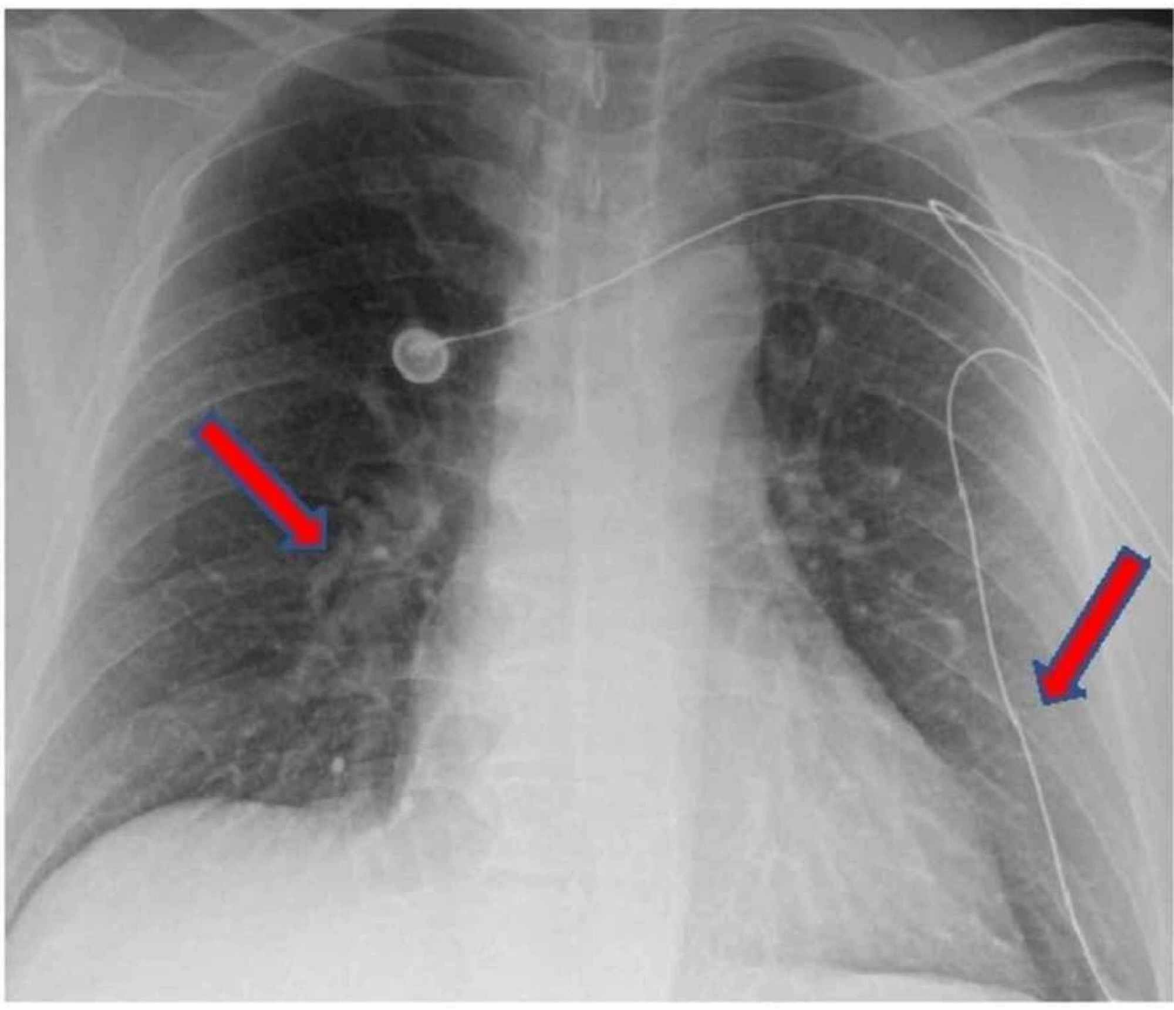
CXR of case 3 showing bilateral patchy infiltrates. CXR: Chest X-ray

CXR of the case 3 is shown in Figure 2.

One patient (case 2) had positive blood culture for Enterococcus faecium. Two patients required intubation during their hospital course. Inflammatory markers (ESR, CRP, IL-6) were elevated in the patients. All three patients were treated with hydroxychloroquine, azithromycin, ceftriaxone for COVID-19 and Oseltamivir for influenza. All were discharged in stable condition. Table 1 summarizes the clinical characteristics of the three patients.

**Table 1:** Summarizes the clinical characteristics and outcome of the three patients. Reference ranges are as follows: white blood cells 4.5-11 K/mm^3^, hemoglobin 12-16 g/dl, platelets 140-440 K/mm^3^, creatine kinase 30-223 unit/L, troponin less than 0.03 ng/ml, sodium 135-145 meq/L, potassium 3.5-5 meq/L, chloride 98-107 meq/L, HCO3 21-31 meq/L, BUN 7-23 mg/dl, creatinine 0.6-1.30 mg/dl, aspartate transaminase 13-39 U/L, alanine transaminase 7-52 U/L, ESR 0-32 mm/hr, CRP less than 10 mg/L, IL-6 0-15.5 pg/ml, fibrinogen 183-503 mg/dl, ferritin 12-300 ng/ml. DM: diabetes mellitus; HTN: hypertension; SOB: shortness of breath; RT-PCR: reverse-transcription polymerase chain reaction; ND: not done; HCO3: bicarbonate; BUN: blood urea nitrogen; CK: creatine kinase; AST: aspartate transaminase; ALT: alanine transaminase; ESR: erythrocyte sedimentation rate; CRP: c-reactive protein; IL-6: Interleukin-6.

Variables	Case 1	Case 2	Case 3
Age	47	71	61
Sex	Male	Female	Female
Race	Pilipino	Hispanic	Hispanic
Medical history	DM	None	DM, HTN
Smoking history	Unknown	Negative	Former
Presenting complaints	Cough, fever	Fever, cough, SOB	SOB, myalgia
Vital signs on presentation: Blood pressure mm Hg / heart rate per minute / temperature degree Celsius / respiratory rate per minute / saturation percent on room air	109-65/88/38/18/98%	196-97/105/35.8 24/83%	175-106/131 37.1/18/94
COVID-19 diagnostic test	RT-PCR	RT-PCR	RT-PCR
Influenza diagnostic test / type	Antigen detection assay/ B	Antigen detection assay/ B	Antigen detection assay/ A
Chest X-ray	Bilateral patchy infiltrates	Multi-lobar infiltrates	Bilateral patchy infiltrates
Chest computed tomography	Diffuse scattered areas of ground-glass and mixed attenuating opacities	Diffuse bilateral ground-glass infiltrates	ND
Blood culture / organism	Negative	Enterococcus faecium	Negative
White blood cells on presentation- K/mm^3^	4.4	15.5	11.5
Hemoglobin- g/dl	15.1	14.4	13.7
Platelets- K/mm^3^	199	246	266
Creatine Kinase total- unit/L	ND	58	343
Troponin- ng/ml	ND	0.04	0.01
Sodium- meq/L	135	141	136
Potassium- meq/L	3.9	3.6	3.9
Chloride- meq/L	102	103	103
HCO3- meq/L	23	24	22
BUN- mg/dl	11	21	11
Creatine- mg/dl	1.23	0.65	0.75
AST/ALT-U/L	31/30	20/15	81/55
ESR- mm/hr	43	38	67
CRP- mg/L	45.8	144.5	203.2
IL-6- pg/ml	62.1	155.2	51
Fibrinogen- mg/dl	ND	546	ND
Ferritin- ng/ml	ND	270	76
Treatment for COVID-19	Hydroxychloroquine, azithromycin and ceftriaxone	Hydroxychloroquine, azithromycin, ceftriaxone, tocilizumab convalescent plasma	Hydroxychloroquine, azithromycin ceftriaxone
Treatment for influenza	Oseltamivir	Oseltamivir	Oseltamivir
Intubation	Yes	Yes	No
Outcome	Discharged	Discharged	Discharged

## Discussion

In a metanalysis study, 3% of patients hospitalized with COVID-19 were also co-infected with another respiratory virus; respiratory syncytial virus (RSV) and influenza A being the most common viral pathogens identified [4]. Table 2 summarizes salient differences between the two viruses [4-7].

**Table 2:** Summarizes salient differences between the two viruses.

	Influenza	COVID-19
Virus characteristics	Influenza viruses are negative-sense single-strand RNA viruses with a segmented genome	Coronaviruses are positive-sense single-stranded, RNA viruses with an unsegmented, genome
Incubation period	1 to 4 days (average 2 days)	Generally, within 14 days following exposure, with most cases occurring approximately four to five days after exposure
Transmission	Respiratory droplets and contact	Respiratory droplets and contact
Diagnostic tests	Antigen detection assays reverse-transcription polymerase chain reaction (RT-PCR), multiplex PCR, and rapid molecular assays	Nucleic acid amplification testing (NAAT) most commonly with RT-PCR assay
Chest X-ray	Bilateral reticular or reticulonodular opacities with or without superimposed consolidation	Consolidation and ground glass opacities
Signs and symptoms	Fever, headache, myalgia, malaise, cough, sore throat, and nasal discharge. Gastrointestinal illness, such as vomiting and diarrhea, is usually not part of influenza infections in adults but can occur in 10 to 20 percent of influenza infections in children.	Fever, cough, dyspnea, nasal discharge, myalgias. However, diarrhea and smell or taste disorders, are also common. Other manifestations include conjunctivitis and dermatologic manifestations - maculopapular, urticarial, and vesicular eruptions and transient livedo reticularis
Laboratory abnormalities	Leukocyte counts are normal or low early in the illness but may become elevated later in the illness	Lymphopenia, elevated aminotransaminase levels, elevated lactate dehydrogenase levels, elevated inflammatory markers (e.g., ferritin, C-reactive protein, and erythrocyte sedimentation rate), and abnormalities in coagulation tests.
Treatment	FDA-approved antiviral drugs	Optimal approach to treatment of COVID-19 is uncertain. Preliminary evidence suggests role for antiviral agent - remdesivir and dexamethasone in the management of COVID-19
Vaccine	FDA-licensed influenza vaccines produced annually	Currently there is no vaccine to prevent COVID-19, clinical trials available
Complications	Acute respiratory distress syndrome (ARDS), myositis, rhabdomyolysis, acute myocardial infarction, myocarditis and pericarditis, toxic-shock syndrome, Guillain–Barre syndrome, transverse myelitis, encephalopathy	Acute respiratory distress syndrome (ARDS), myocarditis, heart failure, acute coronary syndrome, arrhythmias, cardiogenic shock, thromboembolic complications (pulmonary embolism, acute limb ischemia, mesenteric thrombosis, acute stroke), multisystem inflammatory syndrome and Guillain–Barre syndrome.
Bacterial co-infection in intensive care unit (ICU) patients	More prevalent (19% of cases)	Less prevalent (14% of cases)
Most commonly detected bacterial pathogen	Streptococcus pneumoniae, Staphylococcus aureus, Streptococcus pyogenes	Mycoplasma pneumoniae, Pseudomonas aeruginosa, Hemophilus influenzae, Klebsiella pneumoniae

## Conclusions

In conclusion, we report three cases of co-infection of influenza and COVID-19. Health care providers should be aware of this unique situation as both can present with similar symptoms but vary in treatment. Clinicians should have a high index of suspicion in the appropriate clinical scenario. Further studies are needed to determine whether patients who have a concurrent viral infection have a worse prognosis than those in whom SARS-CoV-2 is the only detected pathogen.
